# A nanocarrier containing carboxylic and histamine groups with dual action: acetylcholine hydrolysis and antidote atropine delivery

**DOI:** 10.3762/bjnano.16.2

**Published:** 2025-01-09

**Authors:** Elina E Mansurova, Andrey A Maslennikov, Anna P Lyubina, Alexandra D Voloshina, Irek R Nizameev, Marsil K Kadirov, Anzhela A Mikhailova, Polina V Mikshina, Albina Y Ziganshina, Igor S Antipin

**Affiliations:** 1 A.E. Arbuzov Institute of Organic and Physical Chemistry, FRC Kazan Scientific Centre, Russian Academy of Sciences, Arbuzov str. 8, Kazan 420088, Russia; 2 Alexander Butlerov Institute of Chemistry, Kazan Federal University, Lobachevsky str. 1/29, Kazan 420008, Russia; 3 Kazan National Research Technical University named after A.N. Tupolev - KAI, 10, K. Marx str., Kazan 420111, Russia; 4 Kazan National Research Technological University, 68, K. Marx str., Kazan 420015, Russia; 5 Kazan Institute of Biochemistry and Biophysics FRC Kazan Scientific Centre, Russian Academy of Sciences, Lobachevsky str. 2, Kazan 420111, Russia

**Keywords:** acetylcholine, antidote delivery, artificial cholinesterase, atropine, nanocarrier, resorcinarene

## Abstract

Disruption of cholinesterases and, as a consequence, increased levels of acetylcholine lead to serious disturbances in the functioning of the nervous system, including death. The need for rapid administration of an antidote to restore esterase activity is critical, but practical implementation of this is often difficult. One promising solution may be the development of antidote delivery systems that will release the drug only when acetylcholine levels are elevated. This approach will ensure timely delivery of the antidote and minimize side effects associated with uncontrolled drug release. Here, we describe the creation of a new smart system that serves as a carrier for delivering an antidote (i.e., atropine) and functions as a synthetic esterase to hydrolyze acetylcholine. The nanocarrier was synthesized through microemulsion polycondensation of phenylboronic acid with resorcinarenes containing hydroxy, imidazole, and carboxylic groups on the upper rim. The nanocarrier breaks down acetylcholine into choline and acetic acid. The latter acts on the boronate bonds, dissociating them. This leads to the destruction of the nanocarrier and the release of the antidote. The paper covers the creation of the nanocarrier, its physicochemical and biological properties, encapsulation of the antidote, acetylcholine hydrolysis, and antidote release.

## Introduction

Cholinergic toxicity results from an excessive quantity of acetylcholine (ACh), causing muscle cramps, nausea, vomiting, and other serious issues [1]. ACh overproduction usually results from a malfunction of the cholinesterase enzyme caused by poisoning or medication [2–3]. Drug dosage regulation can greatly lower cholinergic toxicity [4], but the risks associated with poisoning are far higher. Poisons cause permanent disruptions to cholinesterases function. Organophosphorus compounds (OPs) are among these toxins [5]. This class of chemicals finds application in the manufacturing of insecticides, medications, rubber, plastics, paints, and other products. However, if used improperly, the majority of them can have a nerve-paralyzing impact and have major health repercussions. The irreversible binding of OPs to the cholinesterase receptors renders the enzyme completely inactive. An antidote that counters the effects of the OPs on the enzyme is given to treat OP poisoning. Atropine (Atr) [6–8], an alkaloid used to regulate heart rate and treat eye ailments [9–10], is one such antidote. Despite its advantages, Atr has limitations in the treatment of OP poisoning. First, it can be harmful in high doses, and timely administration is vital to prevent permanent damage. Additionally, Atr itself can inhibit cholinesterases, potentially causing increased ACh levels. Developing nanocarriers for Atr delivery could provide a solution. These carriers might improve the efficacy of Atr, prolong its action duration, and reduce its toxicity [11–12].

In recent years, resorcinarenes, which are analogues of calixarenes, have been extensively utilized in the creation of different smart systems [13–14]. Owing to their unique structural features, these macrocycles are employed in catalysis, such as in the breakdown of OPs [15–18], as carriers for drug delivery [19–22], and in the creation of diverse receptors and sensors, notably for ACh [23–27].

**Scheme 1:**
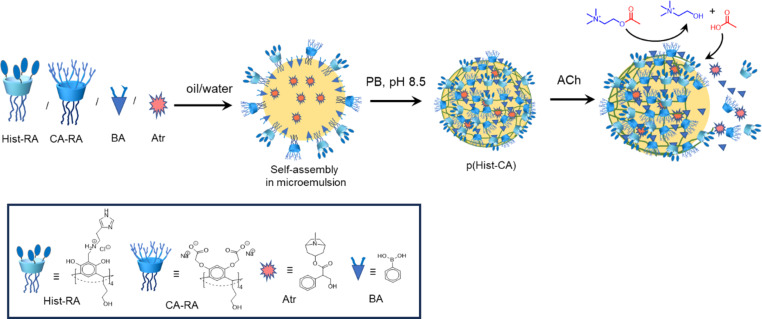
Synthesis of Atr@p(Hist-CA) and Atr release following ACh hydrolysis.

Here, we report the development of a new nanocarrier that can both carry Atr and act as a synthetic esterase to degrade excess ACh. The nanocarrier was synthesized via microemulsion polycondensation of phenylboronic acid with resorcinarenes containing carboxylic, imidazole, and hydroxy groups on the upper rim. Similar to the esterase triad, these groups are essential for the hydrolysis of ACh [28]. Throug hydrolysis, ACh dissociates into choline (Ch) and acetic acid (AcOH). The generated AcOH breaks the boronate bonds and disintegrates the nanocarrier, leading to the release of Atr (Scheme 1). This paper discusses the synthesis of the Atr nanocarrier, its physicochemical and biological properties, the encapsulation of Atr into the nanocarrier cavity, ACh hydrolysis, nanocarrier degradation, and Atr release under the ACh action.

## Results and Discussion

### Synthesis of the nanocarrier p(Hist-CA)

For the development of the Atr nanocarrier, two resorcinarenes were selected, namely, one with carboxylate groups (CA-RA, Scheme 1) and the other with hydroxy and imidazole groups (Hist-RA, Scheme 1). CA-RA was obtained according to [29]. Hist-RA was synthesized by a Mannich reaction from resorcinarene with propanolic groups on the lower rim (RA), formaldehyde, and histamine in two steps, similar to those described in [30–31]. The yield was 41%. The spectral data of CA-RA and Hist-RA are detailed in the Experimental section and are present in Figures S1 and S2 of Supporting Information File 1.

The nanocarrier (p(Hist-CA)) was prepared, using the microemulsion technique [32–33], through the condensation of CA-RA and Hist-RA with phenylboronic acid (BA) in phosphate buffer (PB) at pH 8.5 [34–35]. CA-RA and Hist-RA are amphiphilic molecules. Within a microemulsion system, they self-assemble at the interface between water and oil. The hydrophilic groups face the aqueous phase, while the resorcinarene scaffold with tails on the lower rim points towards the dispersed (oil) phase. Under slightly basic conditions (pH 8.5), resorcinarenes react with BA to form boronate esters through cross-linking (Scheme 1).

**Figure 1:**
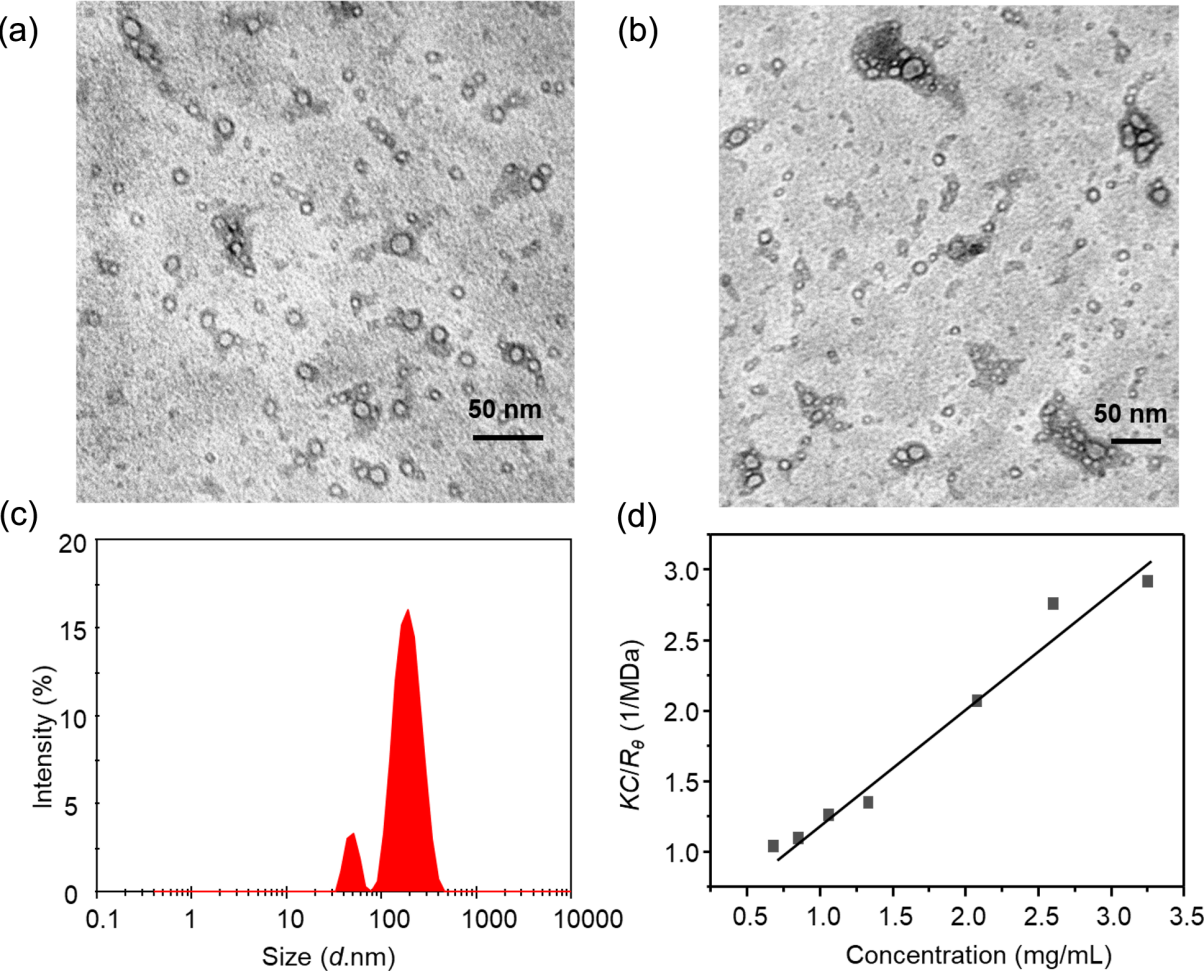
Data for p(Hist-CA): (a, b) TEM images, (c) distribution diagram of the hydrodynamic diameter, *C* = 2 mg/mL and (d) Debye plot, *C* = 0.6–3.3 mg/mL, PB, pH 7.4, 25 °C.

To synthesize p(Hist-CA), 4 mL of BA solution (1.25 mM) in PB, pH 8.5, and 7.3 μL of triolein (TO) were added to a mixture of Hist-RA (2 mM) and CA-RA (4.4 mM) in 1 mL of water. The mixture was vortexed for 1.5 min until an emulsion formed. Subsequently, the emulsion underwent homogenization in an ultrasonic bath while being bubbled with argon for 1.5 h to generate a uniformly dispersed solution. Then, the solution was diluted three times. Polycondensation occurred at 25 °C overnight under stirring. p(Hist-CA) was then purified through dialysis for 1.5 h using a 12000 Da pore dialysis bag. The size of p(Hist-CA) is approximately 12 ± 3 nm according to TEM, and it forms aggregates ranging in size from 80 to 150 nm (Figure 1a,b).

The molecular weight of p(Hist-CA) was determined using gel permeation chromatography (GPC). The GPC profile of the water-soluble portion of the sample showed four peaks (Supporting Information File 1, Figure S3). The main peak, with an average molecular weight of approximately 33 kDa (peak 3, Table 1), was attributed to p(Hist-CA). In the high-molecular-weight region, two low-intensity peaks (peaks 1 and 2) exceeding 2000 kDa likely corresponded to aggregates of p(Hist-CA).

**Table 1:** Molecular weight distribution and polydispersity (PD) for p(Hist-CA) and p(Hist-CA) treated with ACh, *C*(p(Hist-CA) = 1 mg/mL, *C*(ACh) = 4 mM, 0.05 M K_2_HPO_4_, pH 7.4.^a^

Sample	Fraction	Molecular weight averages, kDa					PD

		*M*_p_	*M*_n_	*M*_w_	*M*_z_	*M*_v_	

p(Hist-CA)	peak 1	HM					
	peak 2	HM					
	peak 3	10.1	21.1	32.5	50.9	47.5	1.54
	peak 4	1.1	1.1	1.6	2.8	2.5	1.45

p(Hist-CA) after treatment with ACh	peak 3	2.4	3.8	4.3	5.1	5.0	1.13
	peak 4	0.8	0.8	0.9	1.0	1.0	1.13

^a^HM – high-molecular-weight components, the retention time of which exceeds the calibration curve. *M*_p_ – molecular weight at the peak of the molecular weight distribution curve, *M*_n_ – number-average molecular weight, *M*_w_ – weight-average molecular weight, *M*_z_ – z-average molecular weight, and *M*_v_ – viscosity-average molecular weight.

The particle distribution diagram from DLS data displays two peaks with average hydrodynamic diameters of 50 ± 4 nm and 190 ± 30 nm and with a polydispersity index (PdI) of 0.245 (Figure 1c). Evidently, the former peak corresponds to the hydrodynamic diameter of p(Hist-CA), while the latter one relates to the size of aggregates. The average molecular weight (*M*) of the aggregate was determined using the static light scattering (SLS) Debye plot. The plot of *KC*/*R*_θ_ against *C*, where *K* is the Debye constant, *C* is the concentration, and *R*_θ_ is the Rayleigh ratio, intersects the ordinate at 1/*M*, measuring 0.405 ± 0.125 1/MDa. This implies *M* equals 2750 ± 800 kDa (Figure 1d).

**Figure 2:**
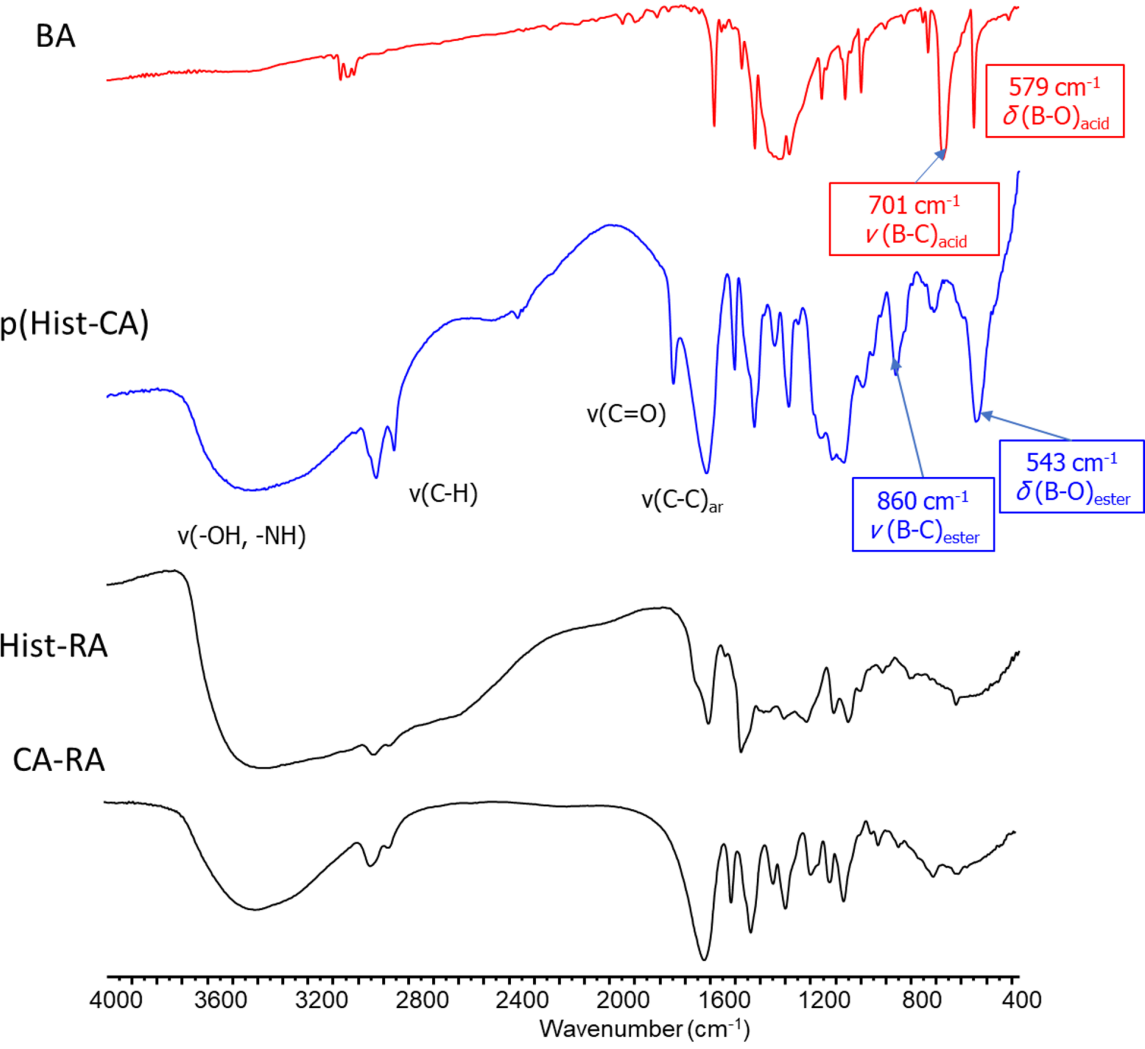
IR spectra of (a) BA, (b) p(Hist-CA), (c) Hist-RA and (d) CA-RA in KBr.

In the IR spectrum, bands of O–H and N–H stretching vibrations of p(Hist-RA) appear at 3700–3100 cm^−1^ (Figure 2). The C–H bond exhibits wild stretching vibration bands at 2700–3000 cm^−1^. A vibration of C=O is fixed at 1744 cm^−1^. Stretching of C–C in aromatic rings is evident at 1610 and 1501 cm^−1^. The p(Hist-RA) IR spectrum also exhibits boronate bond stretching bands at 1066 and 543 cm^−1^, which are not present in the Hist-RA and CA-RA spectra (Figure 2).

### Cytotoxicity and hemocompatibility

According to the cytotoxicity investigation, p(Hist-CA), Hist-RA, and CA-RA were all found to be lowly toxic. Hist-RA showed IC_50_ values greater than 0.22 mM for the WI38 human embryonic lung cell line and the Chang liver human liver cell line (Table 2). The IC_50_ values for CA-RA were 0.57 mM and 0.48 mM for WI38 and Chang liver, respectively. For these cell lines, p(Hist-CA) had an IC_50_ higher than 1.4 mg/mL (Table 2).

**Table 2:** IC_50_ for Hist-RA, CA-RA and p(Hist-CA), obtained on the healthy Chang liver cell line and WI38 human embryonic lung cell line and HC_50_.^a^

	IC_50_		HC_50_

	Chang liver	WI38	

Hist-RA	>220 μM	>220 μM	177 μM
CA-RA	571 ± 48 μM	479 ± 37 μM	>528 μM
p(Hist-CA)	1.48 ± 0.06 mg/mL *C*(Hist-RA) = 196 ± 7 μM *C*(CA-RA) = 392 ± 15 μM	1.75 ± 0.24 mg/mL *C*(Hist-RA) = 232 ± 32 μM *C*(CA-RA) = 464 ± 64 μM	>1.66 mg/mL *C*(Hist-RA) > 220 μM, *C*(CA-RA) > 440 μM

^a^The experiments were performed in triplicate. Results are expressed as the mean ± standard deviation (SD).

**Figure 3:**
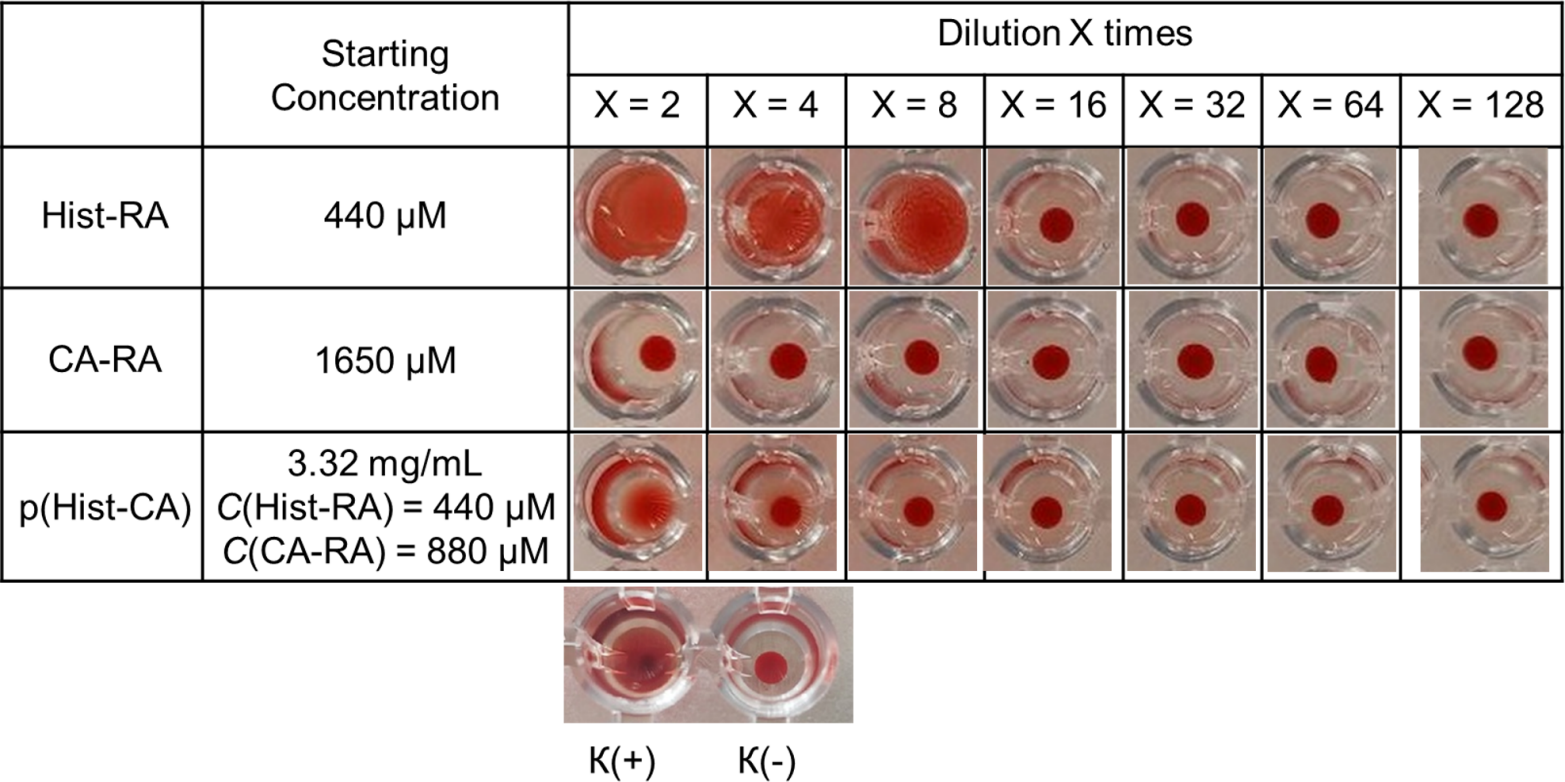
Agglutination assay. Blood samples were observed in the plate wells following the addition of diluted solutions of Hist-RA, CA-RA, and p(Hist-CA) at two- to 128-fold dilutions.

**Figure 4:**
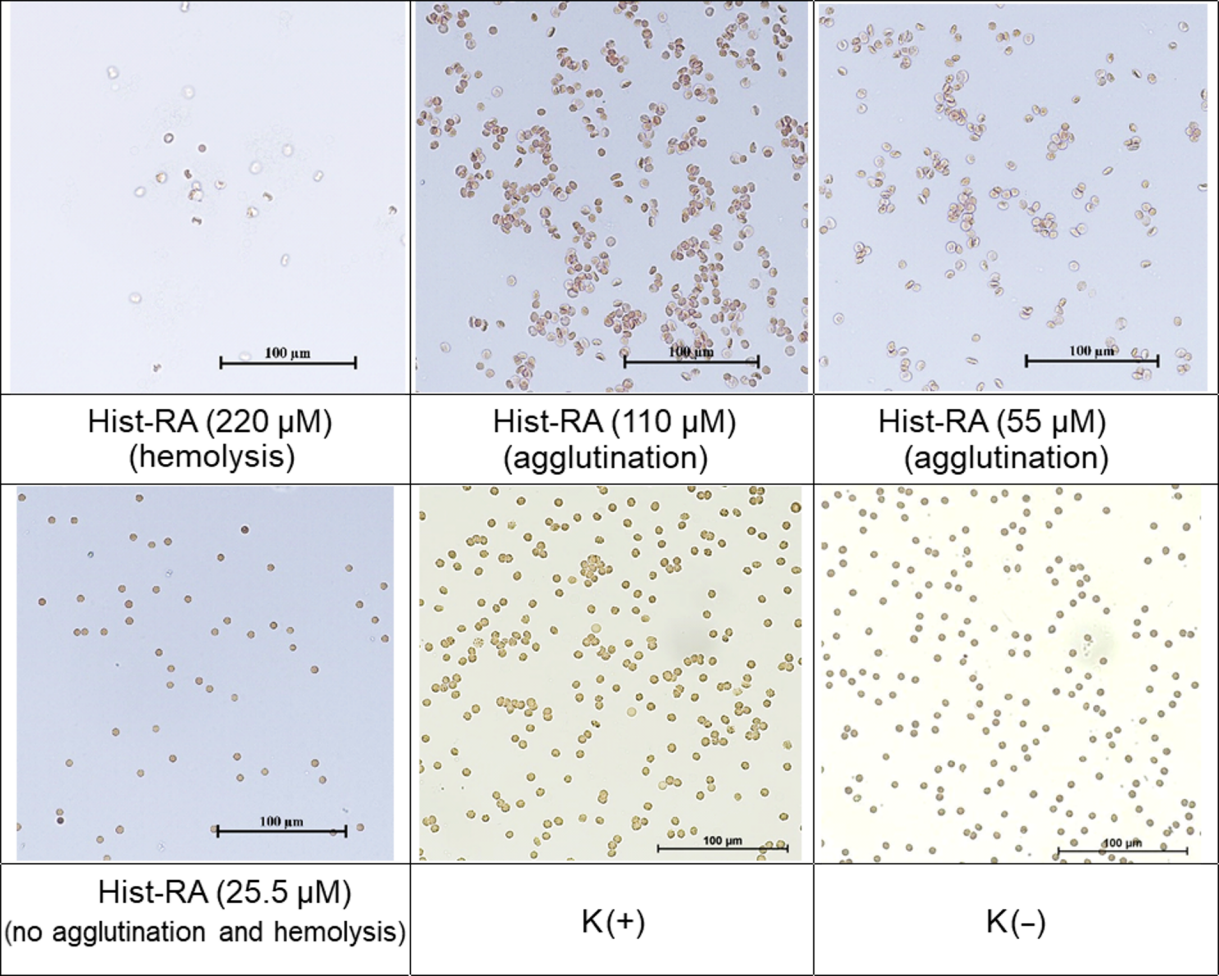
Bright-field microscopy (Nikon Eclipse Ci, 400× magnification). K(−) for intact cells; K(+) for the agglutination control.

The agglutination activity results are shown in Figure 3. Control plate wells with erythrocytes in saline exhibited a dense layer at the bottom, indicating no agglutination (K(−), Figure 3). The positive control (K(+), Figure 3) displayed agglutinated cells (mixture of erythrocytes of blood groups IV and II) evenly distributed in the well. The results revealed that CA-RA exposure to blood samples did not induce agglutination across a wide concentration range (13–1650 μM) and did not cause hemolysis even at the highest concentration of 825 μM (Table 2 and Supporting Information File 1, Figure S4). Hist-RA at 220 μM led to nearly 100% hemolysis (Supporting Information File 1, Figure S4), while at concentrations of 110 and 55 μM, agglutination was observed (Figure 4). With further dilution, no erythrocyte effects were noted, as confirmed by bright-field microscopy (Figure 4). The nanocarrier p(Hist-CA) induced hemolysis (27.1%) and agglutination solely at the highest concentration (Figure 3 and Supporting Information File 1, Figure S4).

### Degradation of p(Hist-CA) under the influence of ACh

The degradation of p(Hist-CA) was analyzed using DLS, GPC, and conductometry. After adding a tenfold excess of ACh compared to the RA fragments, the PdI of p(Hist-CA) increased to 0.6, and three peaks appeared in the distribution diagram with average hydrodynamic diameters of 43 ± 4, 220 ± 130, and 960 ± 250 nm (Supporting Information File 1, Figure S5). This indicates the degradation of p(Hist-CA) in the presence of ACh.

In the GPC curve of p(Hist-CA) treated with ACh, the peaks for high-molecular-weight compounds vanished, revealing peaks with average molecular weights of about 4 kDa and 1 kDa. These were attributed to free resorcinarene derivatives Hist-RA and CA-RA, as well as TO (Table 1, Supporting Information File 1, Figure S3). Additionally, the sample after ACh treatment contained low-molecular-weight components, which included both ACh, its degradation products, and hydrolysis products of phenylboronate esters.

**Figure 5:**
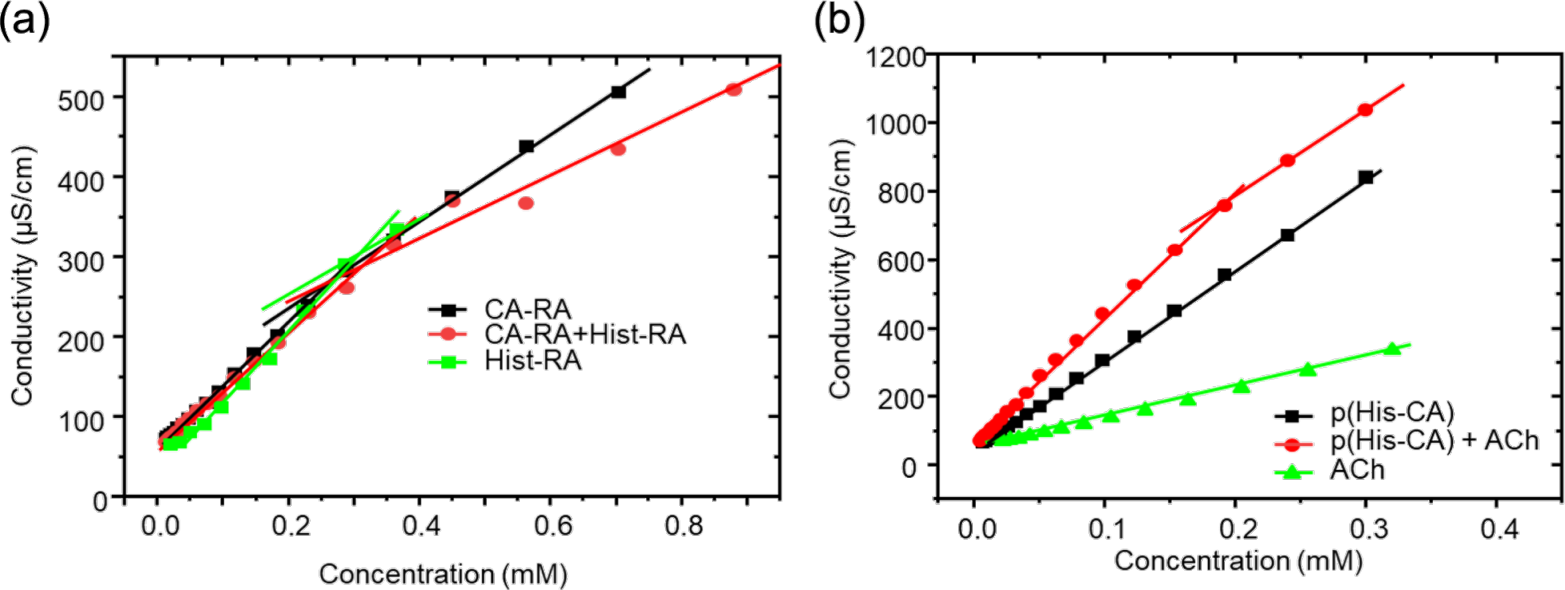
The concentration-dependent conductivity of (a) CA-RA, Hist-RA, and the mixture CA-RA + Hist-RA in H_2_O, 25 °C, and (b) p(Hist-CA), ACh, and the mixture p(Hist-CA) + ACh (1:1) in H_2_O, 25 °C; in the cases of p(Hist-CA) and p(Hist-CA) + ACh, the abscissa represents the RA concentration, while for ACh, it represents the ACh concentration.

Resorcinarenes are known for their self-assembly properties [18]. The electrical conductivity plotted against RA concentration shows a nonlinear relationship for free Hist-RA, CA-RA, and their mixture in water, indicating a critical association constant (CAC) at the break point. The CACs for Hist-RA, CA-RA, and the Hist-RA + CA-RA mixture are 0.34, 0.26, and 0.32 mM, respectively (Figure 5a). In contrast, p(Hist-CA) post-dialysis shows a direct relationship between electrical conductivity and RA fragment concentration, despite its aggregation tendency (Figure 5b). Adding ACh to the p(Hist-CA) solution enhances the electrical conductivity and establishes a break point in the conductivity–concentration curve, with a CAC around 0.2 mM. These results suggest that ACh destabilizes p(Hist-CA), resulting in ensembles of Hist-RA, CA-RA, TO, and boronates, which exhibit a lower CAC than the Hist-RA + CA-RA mixture.

The results from DLS, GPC, and conductometry confirm that p(Hist-CA) is sensitive to ACh and decomposes in its presence, likely due to the hydrolysis of ACh into Ch and acetic acid. This hydrolysis disrupts the boronate bonds, resulting in the decomposition of the polymer.

### Fluorescein encapsulation and release under the influence of ACh

To verify the degradation of p(Hist-CA) by ACh, fluorescein (Fl) was encapsulated into the nanocarrier cavity. Initially, 5 mg of Fl was dissolved in 15–20 mL of ethanol. Next, 7.3 μL of TO was added to the solution, and it was homogenized for 10 min in an ultrasonic bath. The solvent was then removed under reduced pressure. Then, solutions of Hist-RA (4 mM, 0.5 mL, PB, pH 8.5), CA-RA (8.8 mM, 0.5 mL, PB, pH 8.5), and BA (1.25 mM, 4 mL, PB, pH 8.5) were added, and then the synthesis was carried out in a manner similar to that of p(Hist-CA). After the reaction, dialysis was carried out using a 12000 Da dialysis bag to remove unencapsulated Fl, resulting in a solution of Fl@p(Hist-CA). The encapsulation efficiency (%EE) was found to be 55%.

**Figure 6:**
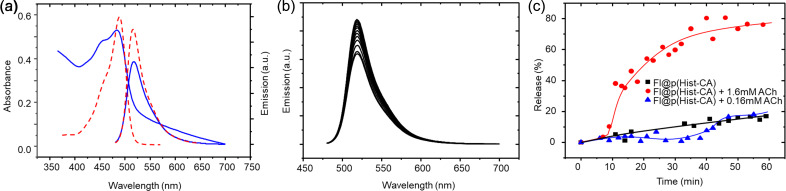
(a) UV and fluorescence spectra of Fl (red dotted line) and Fl@p(Hist-CA) (blue line), (b) fluorescence spectra of Fl@p(Hist-CA) over time following the addition of 1.6 mM ACh, and (c) release of Fl from Fl@p(Hist-CA) over time at 517 nm: without ACh (black squares and line), with 0.16 mM ACh (blue triangles and line), and with 1.6 mM ACh (red circles and line), *C*(Fl) = 0.01 mM; PB, pH 7.4, 37 °C.

In the UV spectra of Fl@p(Hist-CA) (PB, pH 7.4), a hypsochromic shift in absorption typical of Fl in organic solvents is observed, confirming the location of Fl in the organic core. The rise in baseline indicates dispersed media (Figure 6a). In the fluorescence spectra (PB, pH 7.4), the emission intensity of Fl@p(Hist-CA) is significantly lower than that of free Fl because of self-quenching within the confined core and light screening by the polymer shell. Notably, the addition of individual Hist-RA or CA-RA, or of their mixture does not significantly impact Fl emission intensity (Supporting Information File 1, Figure S6a). Similarly, introducing ACh or AcOH, either alone or in combination with Hist-RA or CA-RA, results in a minor increase in emission intensity (1.06 times) (Supporting Information File 1, Figure S6b–e). All of this indicates that Fl is not strongly bound by RA and that the properties of Fl are not affected by the bonding. Fl is also more likely to be found inside p(Hist-CA) than on the surface. The addition of glucose (5 mM) also shows no significant changes, indicating the stability of Fl@p(Hist-CA) under typical glucose and pH conditions.

The addition of ACh to Fl@p(Hist-CA) significantly increases Fl emission (Figure 6b), indicating that Fl is released from the p(Hist-CA) cavity into the bulk. Adding 1.6 mM ACh results in 80% Fl release within 1 h (Figure 6c). In contrast, lowering the ACh concentration to 0.16 mM slows Fl release, yielding only 16% release after 1 h. Similar results were observed without ACh addition, suggesting that 0.16 mM is insufficient to disrupt p(Hist-CA) for effective Fl release.

### Atr encapsulation and release under the influence of ACh

**Figure 7:**
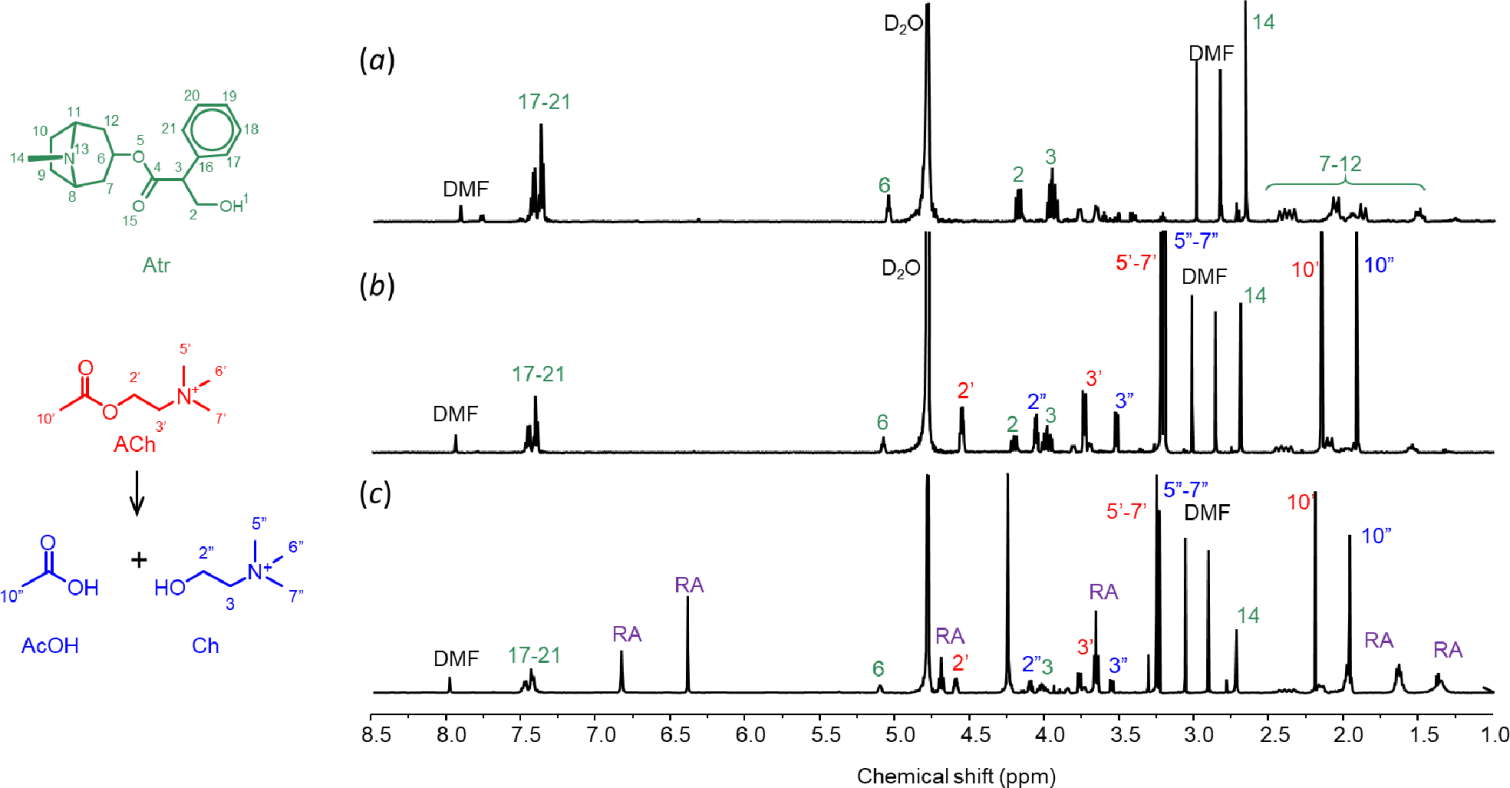
^1^H NMR spectra for Atr@p(Hist-CA): (a) dialysate after synthesis of Atr@p(Hist-CA), (b) dialysate after addition of ACh, and (c) the rest of dialyzing solution, *C*(ACh) = 5 mM, *m*(Atr) = 10 mg, PB, pH 7.4, 25 °C.

Finally, Atr, the antidote for OP poisoning, was inserted into p(Hist-CA). The process involved a reaction similar to that with Fl, but with Atr (10 mg) substituting Fl. Subsequently, polycondensation was carried out following the original procedure. After the polycondensation, the solution underwent dialysis for 1.5 h employing a dialysis bag with 12000 Da pores to eliminate unencapsulated Atr. The resulting dialysate was then distilled under reduced pressure. The quantity of unencapsulated Atr was measured by NMR spectroscopy, applying 0.04% DMF in D_2_O as a reference (Figure 7a). The amount of non-encapsulated Atr was determined by the ratio of the integral of the Atr methyl group signal at 5.05 ppm to the integral of the aldehyde group of DMF at 7.90 ppm. %EE was calculated as 60%.

Then, the polymer particles containing encapsulated Atr (Atr@p(Hist-CA)) were mixed with ACh (5 mM) before undergoing another round of dialysis for 1 h. The dialysate was once again removed under reduced pressure, and the amount of Atr released in response to ACh was evaluated using NMR spectroscopy with 0.04% DMF in D_2_O (Figure 7). The Atr release was 37.5%.

In addition to the signals of released Atr, the ^1^H NMR spectrum includes signals of ACh and its hydrolyzed products, Ch and AcOH. It confirms that ACh indeed undergoes hydrolysis by p(Hist-CA) (Figure 7b). In the ^1^H NMR spectrum of the rest of the dialysis bag, both the signals from the broken-down nanocarrier and the signals of ACh, Ch, and AcOH are present (Figure 7c).

**Figure 8:**
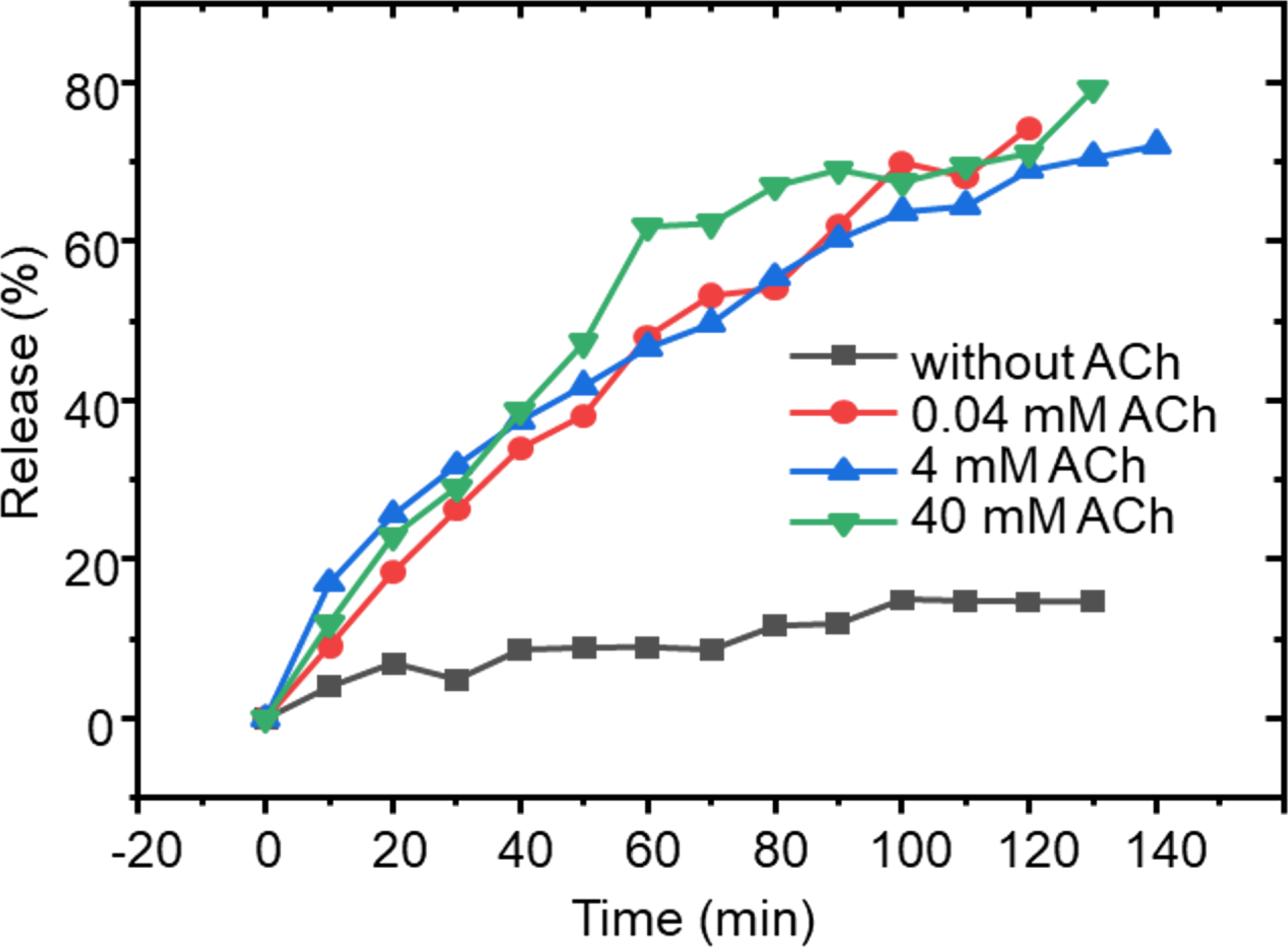
Time-dependent Atr release from Atr@p(Hist-CA) without ACh and after addition of ACh 0.04, 4 and 40 mM in dialyzing experiment, *V*(Atr@p(Hist-CA)) = 3 mL, *V*(dialysate) = 50 mL, PB, pH 7.4.

The yield of Atr over time and varying concentrations of ACh was analyzed using UV spectroscopy (Figure 8). A 3 mL sample of Atr@p(Hist-CA) solution, post-reaction and after 3.5 h of dialysis (*C*(RA) = 0.4 mM), was placed in a 12000 Da dialysis bag with ACh added at concentrations of 0.4, 4, and 40 mM. The dialysate volume was 50 mL. Yield and concentration of atropine were assessed through UV absorption at 224 nm, revealing that approximately 70% of Atr was released after 2 h. Importantly, the release of Atr was not influenced by the concentration of added ACh, suggesting that the release step, rather than the degradation of the carrier, is rate-limiting. Additionally, unlike Fl, even low ACh concentrations facilitate Atr release, indicating that minimal damage to the nanocarrier may be sufficient for Atr to be released.

## Conclusion

A polycondensation reaction of resorcinarenes with caboxylic and imidazole groups with phenylboronic acid in a microemulsion medium was utilized to create a nanocarrier for acetylcholine hydrolysis and antidote delivery. Under healthy conditions, with a neutral pH and normal glucose concentrations, the nanocarrier is found to be stable. The employed resorcinarenes and the nanocarrier exhibit good hemocompatibility and low cytotoxicity with respect to human embryonic lung cells (WI38) and a healthy liver cell line (Chang liver). The nanocarrier catalyzes the hydrolysis of acetylcholine into choline and acetic acid, which causes the dissociation of boronate bonds and the subsequent destruction of the nanocarrier, which was shown by fluorescence and NMR spectroscopy.

## Experimental

### Equipment

Transmission electron microscopy (TEM) images were taken with a Libra 120 EFTEM (A Carl Zeiss SMT AG Company, Oberkochen, Carl Zeiss, Germany) at 100 kV. Samples were spread on a 300 mesh copper grid with a carbon/formvar support film. ^1^H and ^13^C NMR spectra were obtained using a Bruker Avance 600 spectrometer with an operating frequency of 600 MHz. Additionally, IR spectra were recorded using a Bruker Vector 22 Fourier transform spectrometer, in a range from 400 to 4000 cm^−1^ in potassium bromide pellets. Elemental analysis was conducted using a CHNS analyzer EuroEA3028-HT-OM by Eurovector SpA (Italy). Samples were weighed on a Sartorius CP2P microbalance (Germany) in tin capsules. Quantitative measurements and data analysis were performed with Callidus 4.1 software. Hydrodynamic size and molecular weight were analyzed with a ZetaSizer Nano dynamic light scattering photon correlation spectrometer from Malvern, UK. Analysis was performed using Malvern dispersion technology software version 5.10. A Perkin-Elmer Lambda 25 UV–visible spectrometer was utilized for UV spectra collection. Fluorescence spectra were recorded on a HITACHI F 7100 spectrofluorometer. Measurements were conducted at 37 °C using a quartz cuvette that was 1 × 1 × 3.5 cm^3^ in size. The excitation wavelength was 475 nm, and the fluorescence spectra ware measured between 480 and 700 nm. Electronic analytical balances ViBRA AF-225 DRCE (220/0.001 g, resolution 0.00001 g) were utilized for weighing the samples. Ultrasonic treatment was performed with a “Sapfir” UZV 4.0 ultrasonic bath, operating at 280 W power and 35 kHz frequency. Mixing of the samples was conducted using a Multi Speed Vortex MSV-3500 Biosan. PB preparation followed the procedure outlined in [36]. RA was synthesized as described in [37].

The molecular weight and polydispersity indices of p(Hist-CA) (1 mg/mL) and p(Hist-CA) after ACh treatment (4 mM) were analyzed by gel filtration on an Agilent 1260 Infinity chromatography system equipped an OHpak SB-806M HQ (8.0 × 300 mm) column with an OHpak SB-G (6.0 × 50 mm) guard column (Shodex, Japan). The samples were centrifuged prior to use. Elution was carried out using 0.05 M K_2_HPO_4_, pH 7.4, at a flow rate of 0.5 mL·min^−1^ and a column temperature of 40 °C with refractive index detection on a 1260 Infinity detector (Agilent, Germany) at an optical unit temperature of 35 °C. Polyacrylic acid standards of 240, 5, and 1.8 kDa (Alfa Aesar, Acros, and Aldrich, respectively) were used for column calibration. The data was analyzed by Agilent GPC/SEC software.

### Synthesis of Hist-RA

**Scheme 2:**
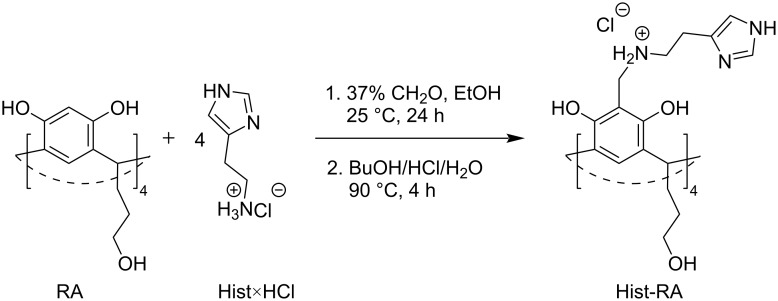
Synthesis of Hist-RA.

Histamine dihydrochloride (Hist×HCl, 0.66 g, 4.47 mmol) was dissolved in 10 mL of double-distilled water and adjusted to pH 10.75. The solvent was evaporated under reduced pressure, and the residue was dissolved in 10–15 mL of ethanol to obtain the Hist solution. In a 100 mL round-bottom flask, RA (0.53 g, 0.74 mmol) was dissolved in 20 mL of ethanol, and a 37% CH_2_O solution (1.79 mL, 22 mmol) was added. Subsequently, the Hist solution was added, and the reaction mixture was stirred at room temperature for 24 h. Following this, the precipitate was filtered and washed with a cold ethanol/water mixture (9/1), which was then dissolved in 15 mL of butanol. After adding 1.5 mL of concentrated hydrochloric acid and 5 mL of double-distilled water, the mixture was stirred at 90 °C for 4 h. The solution was then cooled to room temperature, and 30 mL of chloroform was added. After evaporating the solvent under reduced pressure, 30 mL of diethyl ether was introduced, and the mixture was sonicated for 30 min. The product was subsequently filtered and washed with chloroform multiple times before being dried under reduced pressure at 80 °C for 2 h (Scheme 2). The yield is 0.42 g (42%). ^1^H NMR (600 MHz, D_2_O) δ 1.55 (br., 8H), 2.29 (br., 8H), 3.14 (br., 8H), 3.69 (br., 16H), 4.49 (br., 8H), 5.36 (br., 4H), 7.57 (br., 4H), 8.75 (br., 8H); ^13^C NMR (600 MHz, D_2_O) δ 151, 134, 126, 124, 120, 108, 61, 46, 41, 39, 34, 30, 18; IR (KBr, cm^−1^): 3500–3000, 2938, 1608, 1476; Anal. calcd for C_64_H_88_Cl_4_N_12_O_12_: C, 56.55; H, 6.53; Cl, 10.43; N, 12.37; found: C, 56.48; H, 6.78; Cl, 10.20; N, 11.89.

### Synthesis of CA-RA

**Scheme 3:**
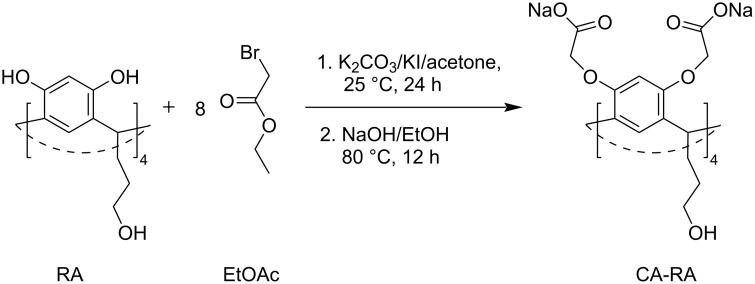
Synthesis of CA-RA.

1. To a solution of 7 g (9.7 mmol) of RA in 100 mL of acetone, 10.74 g (77.6 mmol) of K_2_CO_3_ and 1.6 g (9.7 mmol) of KI were added. The mixture was stirred at room temperature, and a solution of ethyl bromoacetate (13 g, 77.8 mmol) in 100 mL of acetone was added dropwise. After addition, the mixture was stirred at 80 °C for 24 h, followed by the removal of the solvent under reduced pressure (Scheme 3).

2. 50 mL of ethanol and 2 mL of 10 M NaOH were added to the oily residue. The mixture was heated at 80 °C for 12 h. The precipitate was filtered, washed with ethanol, and dried under reduced pressure. The yield is 8.3 g (63%). ^1^H NMR (600 MHz, D_2_O) δ 1.55 (m, 8H), 1.89 (q, 8H), 3.58 (t, 8H), 3.91 (s, 4H), 4.15 (s, 16H), 4.59 (t, 4H), 6.30 (s, 4H), 6.74 (s, 4H); ^13^C NMR (600 MHz, D_2_O) δ 174, 152, 124, 123, 98, 66, 59, 32, 27, 14; IR (KBr, cm^−1^): 2938, 2866, 1608, 1334, 1501, 1423, 834; MALDI-TOF calcd for C_56_H_62_O_28_: 1183; found: 1206 [M + Na + 2H]; 1231 [M + 2Na + 2H]; Anal. calcd for C_56_H_56_O_28_Na_8_: C, 49.42; H, 4.15; Na, 13.51; found: C, 49.00; H, 3.98; Na, 13.91.

### Synthesis of p(Hist-CA)

2.67 mg (2 μmol) of Hist-RA and 5.95 mg (4.4 µmol) of CA-RA were dissolved in 1 mL of double-distilled water. Subsequently, a solution of BA (0.6 mg, 5 μmol) in 4 mL of PB (pH 8.5) was added, along with 7.3 μL of TO. The mixture was vortexed at 3500 rpm for 1 min. Following this, the solution underwent argon purging and 90 min of sonication to homogenize the microemulsion. Then the emulsion was stirred at room temperature overnight. Last, the solution was diluted three times with PB, pH 8.5, and underwent three 30 min dialysis processes using a 12000 Da dialysis bag. IR (KBr, cm^−1^): 3700–3000, 2926, 2856, 1744, 1608, 1476, 1150–1060, 544.

### Synthesis of Fl@p(Hist-CA)

To a Fl (5 mg, 15 μmol) solution in 10 mL of ethanol, 7.3 μL of TO was added, and the mixture was homogenized in an ultrasonic bath. Then ethanol was distilled off under reduced pressure. Hist-RA (2.67 mg, 2 μmol) and CA-RA (5.95 mg, 4.4 µmol) in 1 mL of double-distilled water and BA (0.6 mg, 5 mmol) in 4 mL of PB, pH 8.5, were added. The mixture was vortexed at 3500 rpm for 1 min until an emulsion formed. The solution was then purged with argon in an ultrasonic bath for 90 min. The solution was stirred at room temperature overnight, diluted till 15 mL with PB, pH 8.5, and then purified by dialysis using a 12000 Da dialysis bag.

The amount of encapsulated Fl was determined by UV spectroscopy at 487 nm (ε = 83000 M^−1^·cm^−1^).

%EE was calculated as the ratio of the mass of encapsulated Fl to the initially taken quantity:


[1] $$\%\text{EE}=\frac{m_{\text{Fl}}}{m_{\text{Fl}}^{0}}\times 100\%$$

where *m*_Fl_ is the mass of encapsulated Fl, mg; 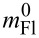 is the mass of Fl taken for the reaction (in mg).

### Synthesis of Atr@p(Hist-CA) and Atr release under ACh influence

Atr@p(Hist-CA) was synthesized, similar to Fl@p(Hist-CA), using 10 mg (25.8 µmol) of atropine sulfate. The resulting Atr@p(Hist-CA) solution underwent purification through a single 1.5 h dialysis process using a 12000 Da dialysis bag. The dialysate was then evaporated under reduced pressure. The residue was dissolved in 650 μL of D_2_O with 0.04% DMF. The amount of unencapsulated Atr was determined by the ratio between the DMF signal intensity at 7.91 ppm and the Atr signal at 5.05 ppm.

ACh (4.54 mg, 25 µmol) was introduced to the purified Atr@p(Hist-CA) solution, followed by another 1.5 h dialysis. The resulting dialysate underwent evaporation under reduced pressure, and the residue was again dissolved in 650 µL of 0.04% DMF in D_2_O. The released Atr amount was also calculated by the ratio between the DMF signal intensity at 7.91 ppm and the Atr signal at 5.05 ppm.

The solvent in the dialyzed solution was evaporated under reduced pressure, and the residue was dissolved in 0.04% DMF in D_2_O. The analysis of the unreleased Atr amount was conducted similarly to the method described in the prior paragraphs.

### Cytotoxicity

Based on fluorescence intensity, the cytotoxic effect was determined using the Cell Viability BioApp on the Cytell Cell Imaging multifunctional system (GE Healthcare Life Science, Sweden) [38]. The experiments utilized a Chang liver cell line (human liver cells) from the N.F. Gamaleya National Research Center for Epidemiology and Microbiology and a cell culture of WI-38 VA 13 subline 2RA (human embryo lung) from the collection of the Institute of Cytology RAS (St. Petersburg, Russia). Standard culture medium Igla, produced by the Moscow Institute of Poliomyelitis and Viral Encephalitis, was employed for cell culture, supplemented with 1% nonessential amino acids (NEAA) and 10% fetal bovine serum. The cells were grown in a CO_2_ incubator at 37 °C after being seeded on a 96-well Eppendorf plate at a concentration of 100,000 cells/mL per well in 150 μL of medium. After 24 h of incubation, 150 μL of the tested dispersions were added to each well. Twofold dilutions of the dispersions were prepared directly in the growth medium.

#### Нemocompatibility

Human erythrocytes were obtained at the medical office of the A.E. Arbuzov Institute of Organic and Physical Chemistry from two Caucasian volunteers (52 years old, blood group II and 24 years old, blood group IV), after receiving informed consent. The study was approved by the ethical committee of the FRC Kazan Scientific Center of RAS according to Russian national ethical guidelines (protocol No. 9–2013). No organs/tissues were procured from prisoners.

#### Hemolysis of human red blood cells

The hemolytic activity of Hist-RA, CA-RA, and p(Hist-CA) were evaluated against human red blood cells (hRBCs) [38]. Following a 10 min, 800 rpm centrifugation of fresh hRBCs with EDTA, the cells were washed three times with PBS (35 mM PB/0.15 M NaCl, pH 7.3) before being resuspended in PBS. Then, 0.5 mL of a hRBC stock solution in PBS was mixed with samples dissolved in PBS to reach a final volume of 5 mL (final erythrocyte concentration, 10% v/v). The resultant suspension was agitated and incubated at 37 °C for 1 h. After that, the samples were centrifuged for 10 min at 2000 rpm. The absorbance of the supernatant at 540 nm was used to calculate the amount of hemoglobin that had been liberated. Controls for zero hemolysis (blank) and 100% hemolysis consisted of hRBCs suspended in PBS and distilled water, respectively. The experiments were repeated three times.

#### Hemagglutination test

Human erythrocytes of blood groups II and IV were utilized in the study. The erythrocytes underwent two washes with 0.9% saline solution and were centrifuged at 2500*g* for 10 min at 4 °C. Following each centrifugation cycle, the supernatant was meticulously discarded. Subsequently, the red blood cells were resuspended in 0.9% saline to achieve a concentration of 2%. The hemagglutination activity of the test compounds was examined in a 96-well U-microtiter plate. Subsequently, two-fold serial dilutions of the studied compositions were prepared. 100 μL of each dilution was combined with 100 μL of 2% packed red blood cells (1:1) and added to a well of a 96-well U microtiter plate. Each dilution was tested in two parallel wells. The samples were then incubated for 1 h at 37 °C, followed by hemagglutination observation with the naked eye (Figure 3) [39]. Images of the samples were captured using a Nikon Eclipse Ci-S microscope (Nikon, Japan) (Figure 4).

## Supporting Information

File 1 ^1^H and ^13^C NMR spectra of Hist-RA and CA-RA; GPC data (molecular weight distribution); hemolytic activity of Hist-RA, CA-RA, and p(Hist-CA); DLS data of p(Hist-CA) before and after ACh addition; and the effects of CA-RA, Hist-RA, ACh, and AcOH on the fluorescence spectrum of Fl.

## Data Availability

The data that supports the findings of this study is available from the corresponding author upon reasonable request.
