# Supplementary material for: A nanocarrier containing carboxylic and histamine groups with dual action: acetylcholine hydrolysis and antidote atropine delivery
Source: Beilstein J Nanotechnol. 2025 Jan 9;16:11–24. doi: 10.3762/bjnano.16.2 (PMC11729679; doi:10.3762/bjnano.16.2)
Supplement: File 1 — 1H and 13C NMR spectra of Hist-RA and CA-RA; GPC data (molecular weight distribution); hemolytic activity of Hist-RA, CA-RA, and p(Hist-CA); DLS data of p(Hist-CA) before and after ACh addition; and the effects of CA-RA, Hist-RA, ACh, and AcOH on the fluorescence spectrum of Fl. [file Beilstein_J_Nanotechnol-16-11-s001.pdf]

BEILSTEIN JOURNAL OF NANOTECHNOLOGY

Supporting Information

for

A nanocarrier containing carboxylic and histamine groups with dual action: acetylcholine hydrolysis and antidote atropine delivery

Elina E. Mansurova, Andrey A. Maslennikov, Anna P. Lyubina, Alexandra D. Voloshina, Irek R. Nizameev, Marsil K. Kadirov, Anzhela A. Mikhailova, Polina V. Mikshina, Albina Y. Ziganshina and Igor S. Antipin

Beilstein J. Nanotechnol. **2025,** *16,* 11–24. doi:10.3762/bjnano.16.2

^{1}H and ^{13}C NMR spectra of Hist-RA and CA-RA; GPC data (molecular weight distribution); hemolytic activity of Hist-RA, CA-RA, and p(Hist-CA); DLS data of p(Hist-CA) before and after ACh addition; and the effects of CA-RA, Hist-RA, ACh, and AcOH on the fluorescence spectrum of Fl

License and Terms: This is a supporting information file under the terms of the Creative Commons Attribution License (https://creativecommons.org/licenses/by/4.0). Please note that the reuse, redistribution and reproduction in particular requires that the author(s) and source are credited and that individual graphics may be subject to special legal provisions.
The license is subject to the *Beilstein Journal of Nanotechnology* terms and conditions: (https://www.beilstein-journals.org/bjnano/terms)

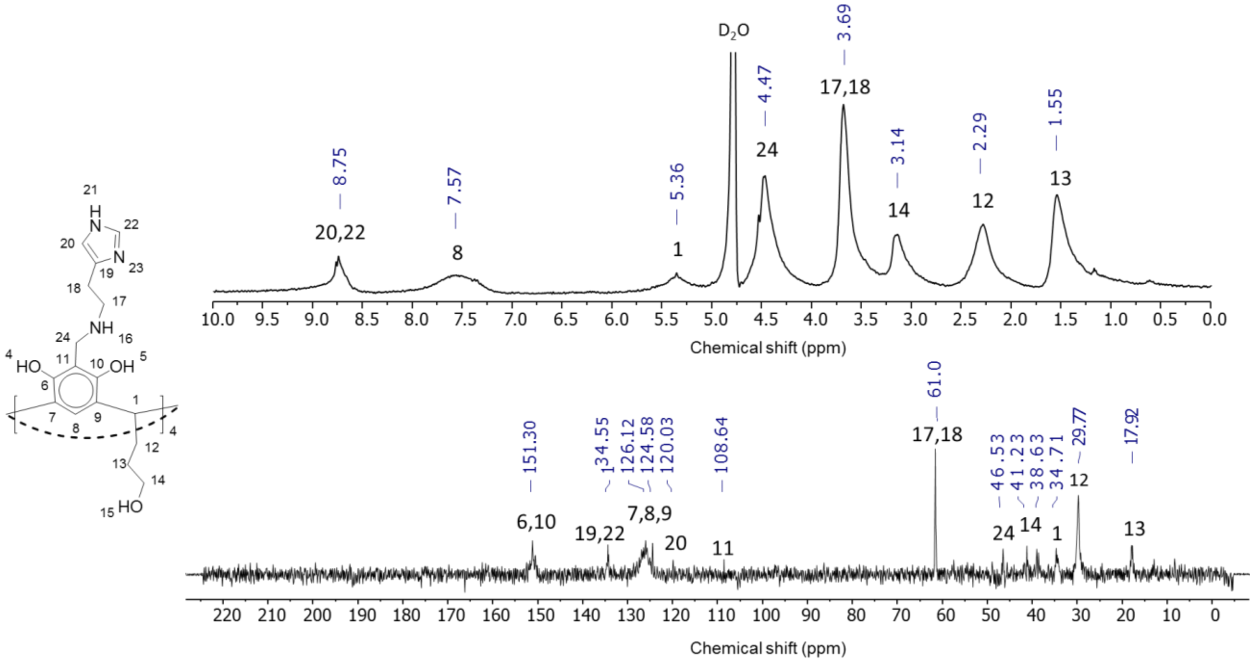

Figure S1: ^{1}H and ^{13}C NMR spectra of Hist-RA in D_2O, 25 °C.

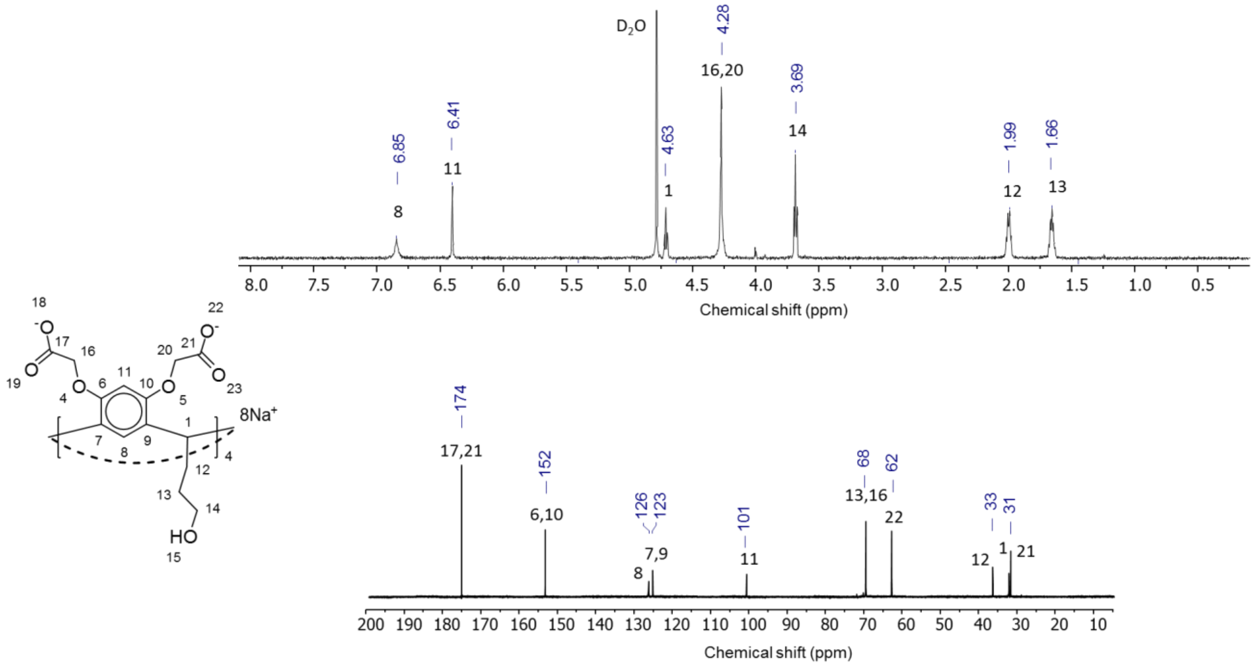

Figure S2: ^{1}H and ^{13}C NMR spectra of CA-RA in D_2O, 25 °C.

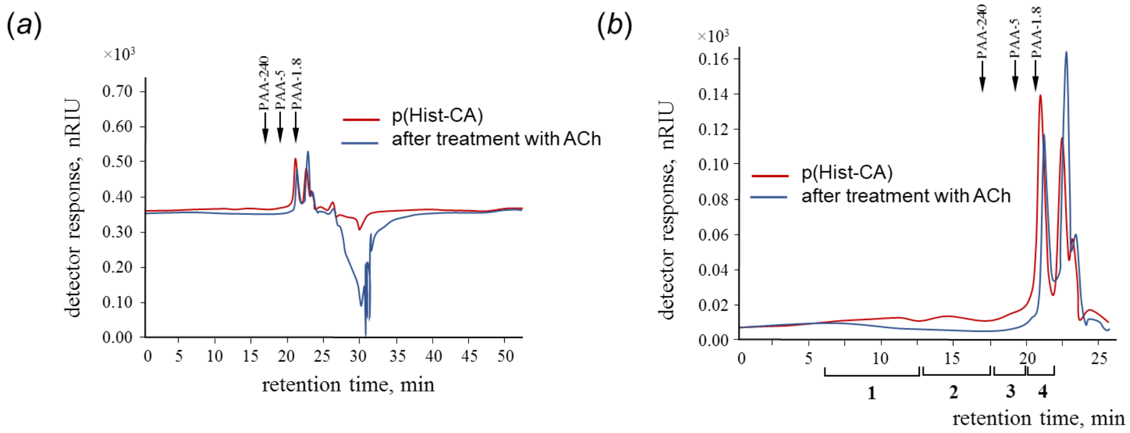

Figure S3: Molecular weight distribution of water-soluble portion of p(Hist-CA) before and after addition of ACh at separation on OHpak SB-806M HQ column. Combinations of peaks are indicated by brackets, (a) 50 min period, (b) 25 min period, *C*(p(Hist-CA) = 1 mg/mL, *C*(ACh) = 4 mM, 0.05 M K_2HPO_4, pH 7.4.

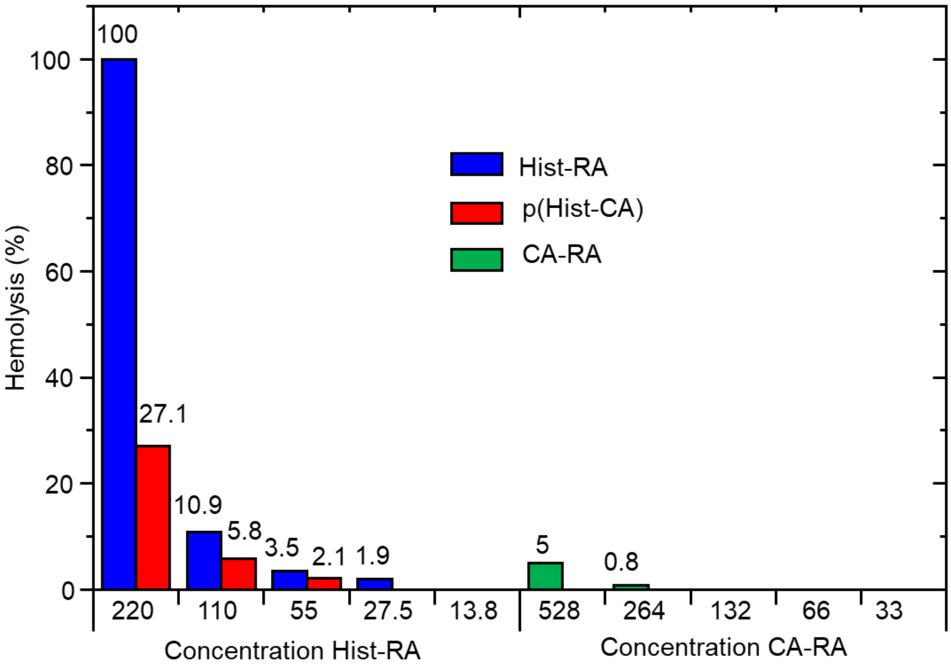

Figure S4: The dependance of hemolysis (%) on the concentration of Hist-RA, CA-RA and p(Hist-CA).

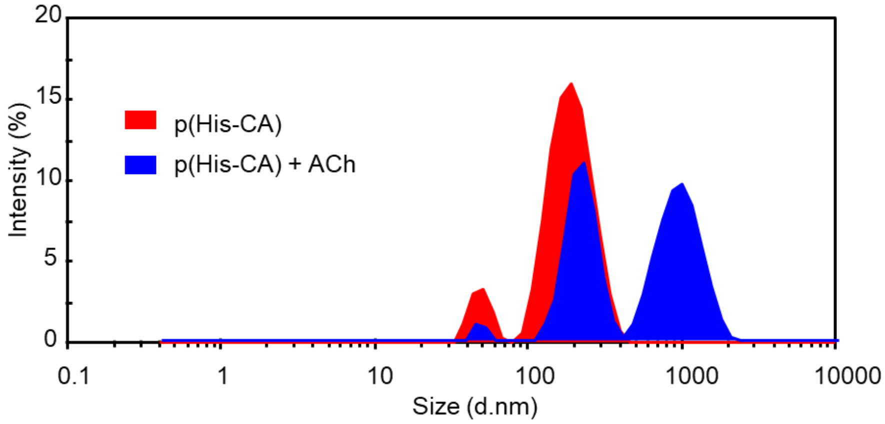

Figure S5: Distribution diagrams of the hydrodynamic diameter of p(Hist-CA) before and after addition of ACh, C(p(Hist-CA)) = 1 mg/mL, C(ACh) = 4 mM, PB pH 7.4, 25 °C.

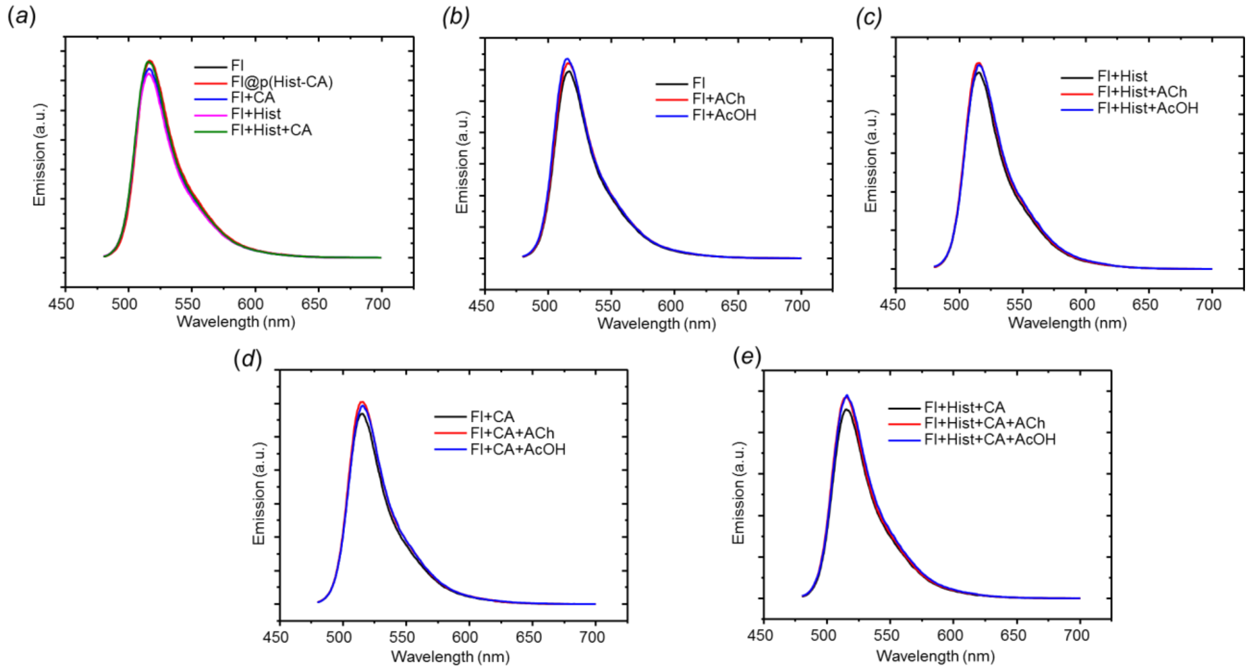

Figure S6: Fluorescence spectra of (a) Fl, Fl@p(Hist-CA) and mixtures of Fl + CA-RA, Fl + Hist-RA, Fl + Hist-RA + CA-RA, (b–e) systems before and after addition of ACh or AcOH: (b) Fl, (c) Fl + Hist-RA, (d) Fl + CA-RA and (e) Fl + Hist-RA + CA-RA, C(Fl) = 0.01 mM; C(Hist-RA) = 0.05 mM, C(CA-RA) = 0.11 mM, C(ACh) = C(AcOH) = 1.6 mM; PB pH 7.4, 25 °C.
